# LIM homeobox protein 5 (Lhx5) is essential for mamillary body development

**DOI:** 10.3389/fnana.2015.00136

**Published:** 2015-10-27

**Authors:** Amaya Miquelajáuregui, Teresa Sandoval-Schaefer, Miriam Martínez-Armenta, Leonor Pérez-Martínez, Alfonso Cárabez, Yangu Zhao, Michael Heide, Gonzalo Alvarez-Bolado, Alfredo Varela-Echavarría

**Affiliations:** ^1^Departamento de Neurobiología del Desarrollo y Neurofisiología, Instituto de Neurobiología, Universidad Nacional Autónoma de MéxicoQuerétaro, Mexico; ^2^Laboratorio de Neuroinmunobiología, Departamento de Medicina Molecular y Bioprocesos, Instituto de Biotecnología, Universidad Nacional Autónoma de MéxicoCuernavaca, Mexico; ^3^Program on Genomics of Differentiation, Eunice Kennedy Shriver National Institute of Child Health and Human Development, National Institutes of HealthBethesda, MD, USA; ^4^Institute of Anatomy and Cell Biology, University of HeidelbergHeidelberg, Germany

**Keywords:** transcription factor, diencephalon, hypothalamus, mouse, embryonic development

## Abstract

The mamillary body (MM) is a group of hypothalamic nuclei related to memory and spatial navigation that interconnects hippocampal, thalamic, and tegmental regions. Here we demonstrate that *Lhx5*, a LIM-HD domain transcription factor expressed early in the developing posterior hypothalamus, is required for the generation of the MM and its derived tracts. The MM markers *Foxb1*, *Sim2*, and *Lhx1* are absent in Lhx5 knock-out mice from early embryonic stages, suggesting abnormal specification of this region. This was supported by the absence of *Nkx2.1* and expansion of *Shh* in the prospective mamillary area. Interestingly, we also found an ectopic domain expressing *Lhx2* and *Lhx9* along the anterio-posterior hypothalamic axis. Our results suggest that Lhx5 controls early aspects of hypothalamic development by regulating gene expression and cellular specification in the prospective MM.

## Introduction

The hypothalamus is an important regulator of endocrine, autonomic, and behavioral processes. Additional functions in memory and spatial navigation have been proposed for the mamillary body (MM), a group of nuclei in the posterior hypothalamus that form part of an extended limbic system, the “Papez circuit” (Papez, 1937; Vann and Aggleton, 2004). The MM receives direct inputs from the hippocampal formation via the fornix and projects to the anterior thalamic nuclei through the mammillothalamic tract (mtt). The MM also forms reciprocal connections with the tegmental nuclei of Gudden, with afferents and efferents via the mamillary peduncle and mammillotegmental (mteg) tract, respectively (for review, see Dillingham et al., 2014). In humans, atrophy of the MM has been associated with poor memory recall (Tsivilis et al., 2008) and patients with “obstructive sleep apnea” or “amnesic Korsakoff’s syndrome” display both memory deficits and reduced MM volume, the latter concurrent with anterior thalamic pathology (Vann and Aggleton, 2004; Kumar et al., 2008; Kril and Harper, 2012). Supporting a role for the MM in memory, a recent study in rats demonstrated that lesions to either mtt outputs or ventral tegmental inputs (but not to hippocampal inputs) lead to short-term memory impairments (Vann, 2013). In addition, the lateral nucleus of the MM is a key component of the head direction (HD) signal generator and believed to be essential for spatial orientation (Clark and Taube, 2012).

The MM is located at the posteroventral hypothalamus. Most MM neurons are generated in the neuroepithelium of the mamillary recess in an “outside-in”, dorso-lateral to ventro-medial neurogenetic gradient, between embryonic (E) days 10 to 16 in the mouse (Shimada and Nakamura, 1973; Shimogori et al., 2010), although neurons born as early as E8.5 from *Shh*^+^ progenitors have also been detected (Alvarez-Bolado et al., 2012).

Cellular and regional identities in the hypothalamus are instructed by morphogens and transcription factors that are highly conserved through vertebrate evolution (Shimamura et al., 1995; Puelles et al., 2000; Blackshaw et al., 2010; Pearson and Placzek, 2013). The induction and early patterning of the basal and anterior hypothalamus, requires the activity of Shh and dynamic crosstalk between BMP and Wnt signaling to control gene expression (Kimura et al., 1996; Mathieu et al., 2002; Manning et al., 2006; Vieira and Martinez, 2006; Szabo et al., 2009a; Shimogori et al., 2010). For example, the transcription factor *Nkx2.1* (previously *T/ebp*), an early marker of basal hypothalamus, is crucial for ventral hypothalamic patterning (Kimura et al., 1996; Puelles et al., 2000; Shimogori et al., 2010). Notably, expression of *Foxb1* (previously *Fkh5*) is confined to the MM throughout mouse hypothalamic development, and is required for the maintenance of its medial component and the formation of the mtt, although not for MM generation *per se* (Wehr et al., 1997; Alvarez-Bolado et al., 2000). Similarly, in compound Sim1/Sim2 knockout mice, the MM is present, but its mteg and mtt efferent tracts fail to form (Marion et al., 2005).

The spatio-temporal expression patterns of the LIM homeobox family of transcription factors delineate different anatomical compartments of the developing CNS in vertebrates (Hobert and Westphal, 2000; Abellan and Medina, 2009; Shimogori et al., 2010). Interestingly, embryonic expression of *Lhx1/5* and *Lhx2/9* subgroups in alternating diencephalic (Retaux et al., 1999; Bachy et al., 2001) suggests that positive and negative interactions between members of the family help orchestrate regional specification, as observed in the spinal cord and cerebellum (Hobert and Westphal, 2000; Jessell, 2000; Pillai et al., 2007; Zhao et al., 2007b). We have previously reported that *Lhx5* is essential for hippocampal morphogenesis (Zhao et al., 1999), for the development of subsets of hindbrain reticulospinal neurons (Cepeda-Nieto et al., 2005) and for the specification and migration of Cajal–Retzius neurons in the telencephalon (Miquelajauregui et al., 2010). In this study, we demonstrate in mice that *Lhx5* is a key factor in posterior hypothalamic specification and that it is required for the formation of the MM and associated tracts.

## Materials and Methods

### Animals

Lhx5-null (Lhx5^-/-^) mice were maintained in a CD-1 background and genotyped by PCR as described before (Zhao et al., 1999). Controls were either wild-type or heterozygous littermates, and at least three embryos were analyzed per condition. The day of detection of the vaginal plug was designated embryonic day (E) 0.5. Pregnant females were euthanized with CO_2_ by trained personnel with a minimum of distress for the animals. Animals were housed and handled in compliance with National Institutes of Health regulations, Mexican governmental guidelines regarding the use of laboratory animals for research purposes (NOM-062-ZOO-1999) and following the “Guide for Care and use of laboratory animals” of the Institute of Laboratory Resources, National Research Council. The work in this study was approved by the Research Ethics Committee (“Comité de Ética en Investigación”), of the Instituto de Neurobiología, UNAM.

### Tissue Preparation

Embryos were fixed in 4% paraformaldehyde (PFA) in PBS (pH 7.4) for 16 h at 4°C, thoroughly washed in PBS and dissected. To prepare frozen sections, tissue was cryoprotected by submersion in 30% sucrose in PBS for 16 h and embedded in Tissue Tek OCT compound (Miles, Elkhart, IN, USA). Coronal sections (10 μm) were obtained and mounted on Superfrost-plus slides (Thermo-Fisher Scientific, Waltham, MA, USA), dried for 30 min and stored at -70°C.

### Histochemistry

Fixed brains were dehydrated, embedded in paraffin, and sectioned (20 μm). Tissue was rehydrated and processed for Nissl staining following standard protocols. To label axonal tracts we used the Sevier–Munger silver staining method (Sevier and Munger, 1965; Chaplin, 1985). Briefly, sections were incubated in a 20% silver nitrate solution in water for 15 min at 60°C. After individually rinsed in water, slides were placed in ammoniacal silver solution (see below) for 5–30 min and developed with gentle stirring until golden brown. Slides were then rinsed in three changes of water and placed in 5% sodium thiosulfate for 2 min, dehydrated in two changes each of 95% ethanol, absolute ethanol and xylene and mounted with Permount (Thermo-Fisher Scientific, Waltham, MA, USA). Ammoniacal silver solution was prepared fresh by adding dropwise to 50 ml of 10% (w/v) silver nitrate the following while stirring: 30% ammonium hydroxide until the dark brown precipitate that forms disappears almost completely, 0.5 ml of 1% sodium carbonate, and 25 drops of 30% ammonium hydroxide followed by filtration.

### *In Situ* Hybridization (ISH)

Chromogenic *in situ* hybridization (ISH) was performed in whole-mount preparations, as described elsewhere (Varela-Echavarria et al., 1996). Digoxigenin (DIG)-labeled antisense riboprobes were synthesized by *in vitro* transcription using cDNA templates. The following plasmids were used: Lhx5 (Zhao et al., 1999); Lhx1 (Miquelajauregui et al., 2010); Sim2, Nkx2.1, and Foxb1 (Marion et al., 2005); Lhx2 and Lhx9 (Bertuzzi et al., 1999). Shh, Tbr1 (IMAGE clone 6817237, Invitrogen). For Supplementary Figure S1, the following data from the Allen Developing Mouse Brain Atlas (http://developingmouse.brain-map.org) was used: Lhx5 E11.5 (GI: 31982215, NM_008499.2, Image No. 100028591.43); Lhx5 E13.5 (GI: 31982215, NM_008499.2, Image No.100026515.65); Irx5 E11.5 (GI: 42476078, NM_018826.2, Image No. 100072726.61); Lmx1b E13.5 (GI: 6754561, NM_010725.1, Image No. 100047108.67).

### Nomenclature

The nomenclature used in the present study generally follows that proposed by (Shimogori et al., 2010), taking into account the prosomeric model (Puelles et al., 2012).

## Results

### *LHX5* is Expressed in the Prospective MM

**FIGURE 1:**
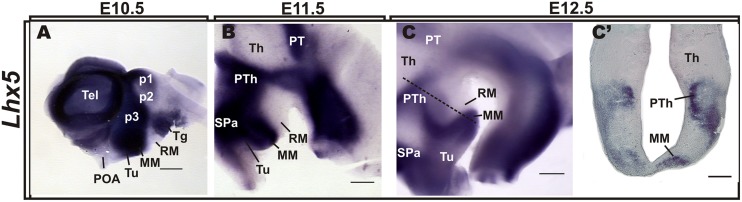
**Expression patterns of *Lhx5* in the developing hypothalamus.** *In situ* hybridization for *Lhx5* in whole-mount brain preparations of E10.5 **(A)**, E11.5 **(B),** and E12.5 **(C)** embryos and in a section indicated in **(C)** by the dashed line **(C’)**. Whole hybridized brains were hemisected along the sagittal plane and lateral views are shown (anterior is to the left). Abbreviations: Th, thalamus; MM, mamillary body; p, prosomere; POA, preoptic area; PT, pretectum; PTh, prethalamus; RM, retromamillary; SPa, subparaventricular area; Tel, telencephalon; Tg, tegmentum; Tu, tuberal. Scale bars: **(A–C)** 400 μm, **(C’)** 200 μm.

We analyzed the pattern of *Lhx5* expression in the mouse hypothalamic area at E10.5–12.5, around the peak of neuron generation in the prospective MM (Shimada and Nakamura, 1973; Altman and Bayer, 1986), (**Figure 1**). At E10.5, *Lhx5* expression was mainly found in the basal hypothalamus and was particularly strong in posterior regions, including the prospective MM. Later, at E11.5 and 12.5, *Lhx5* expression in the hypothalamus receded and remained strong in mamillary areas, although low levels of expression were also found in the abutting tuberal hypothalamic region (**Figures 1B,C**).

In agreement with previous work, *Lhx5* also labeled p3 (prethalamus, PTh) and p1 (pretectum, PT), in addition to the subparaventricular area (SPa), the thalamic eminence (eminentia thalami; EMT), the tegmentum, and the zona limitans intrathalamica (ZLI), whereas p2 (thalamus, Th) and presumptive retromamillary (RM) and suprachiasmatic (SCN) regions appeared devoid of expression (**Figure 1** and Supplementary Figure S1), as previously described (Sheng et al., 1997; Retaux et al., 1999; Bachy et al., 2001; Abellan and Medina, 2009). Notably, *Lhx5* expression abuts the *Lmx1b* and *Irx5*^+^ RM (also supramammillary) region at E11.5 and E13.5, respectively, in a somewhat complementary manner (Supplementary Figure S1), suggesting that *Lhx5* is not expressed in the RM.

Whereas early *Lhx5* expression at early stages spans the entire thickness of the hypothalamic primordium (ventricular zone and incipient mantle layer; Sheng et al., 1997, Heide et al., 2015 and data not shown), analysis of coronal sections at E12.5 indicates that *Lhx5* is expressed mostly, if not exclusively, in mantle regions (**Figure 1C**’). This suggests that *Lhx5* in the hypothalamus is initially expressed in both mitotic cells and post-mitotic neurons but persists in early post-mitotic neurons at later stages, as reported in the developing cortex, spinal cord, retina, and cerebellum (Cepeda-Nieto et al., 2005; Pillai et al., 2007; Zhao et al., 2007b; Miquelajauregui et al., 2010).

### The MM and its Main Tracts are Absent in *LHX5* Knock-out Mice

**FIGURE 2:**
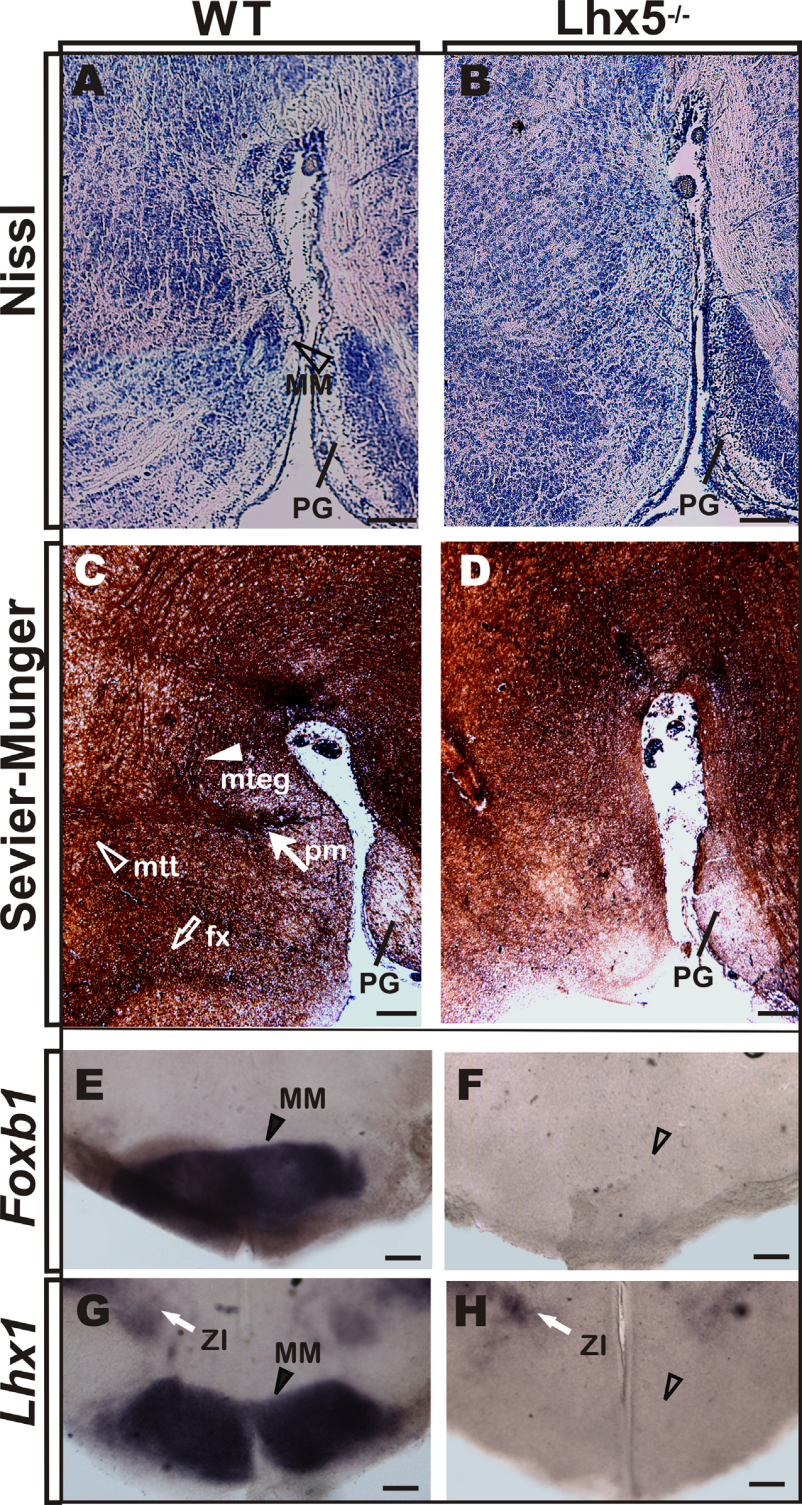
**MM is absent in Lhx5 mutants. (A,B)** Nissl staining in sagittal sections of control mice at E18.5 reveals the MM (arrowhead) that appears absent in Lhx5 mutants. **(C,D)** Comparable sections processed for Sevier–Munger silver staining reveal the fx (empty arrow), pm (filled arrow), mtt, (empty arrowhead), and mteg (filled arrowhead) tracts, in control but not in mutant littermates (anterior is to the left). **(E–H)** *In situ* hybridization for *Foxb1* and *Lhx1* in E18.5 coronal sections stained the MM in controls (arrowhead) and showed no expression in Lhx5 mutants. Note the presence of *Lhx1* expression in the ZI (white arrow). Abbreviations: fx, fornix; MM, mamillary; mteg, mammillotegmental; mtt, mammillothalamic; PG, pontine gray; pm, principal mamillary; ZI, zona incerta. Scale bar: 400 μm.

To determine the role of *Lhx5* in the development of the MM, we analyzed knock-out mice lacking *Lhx5* function (Zhao et al., 1999). Since absence of *Lhx5* leads to perinatal lethality, potentially caused by breathing-control deficiencies associated with early hindbrain specification (Sheng et al., 1997; Cepeda-Nieto et al., 2005), we performed histological analyses on E18.5 embryos, when the development of the MM and its main axonal tracts is complete (Alvarez-Bolado et al., 2000; Marion et al., 2005), (**Figure 2**).

**FIGURE 3:**
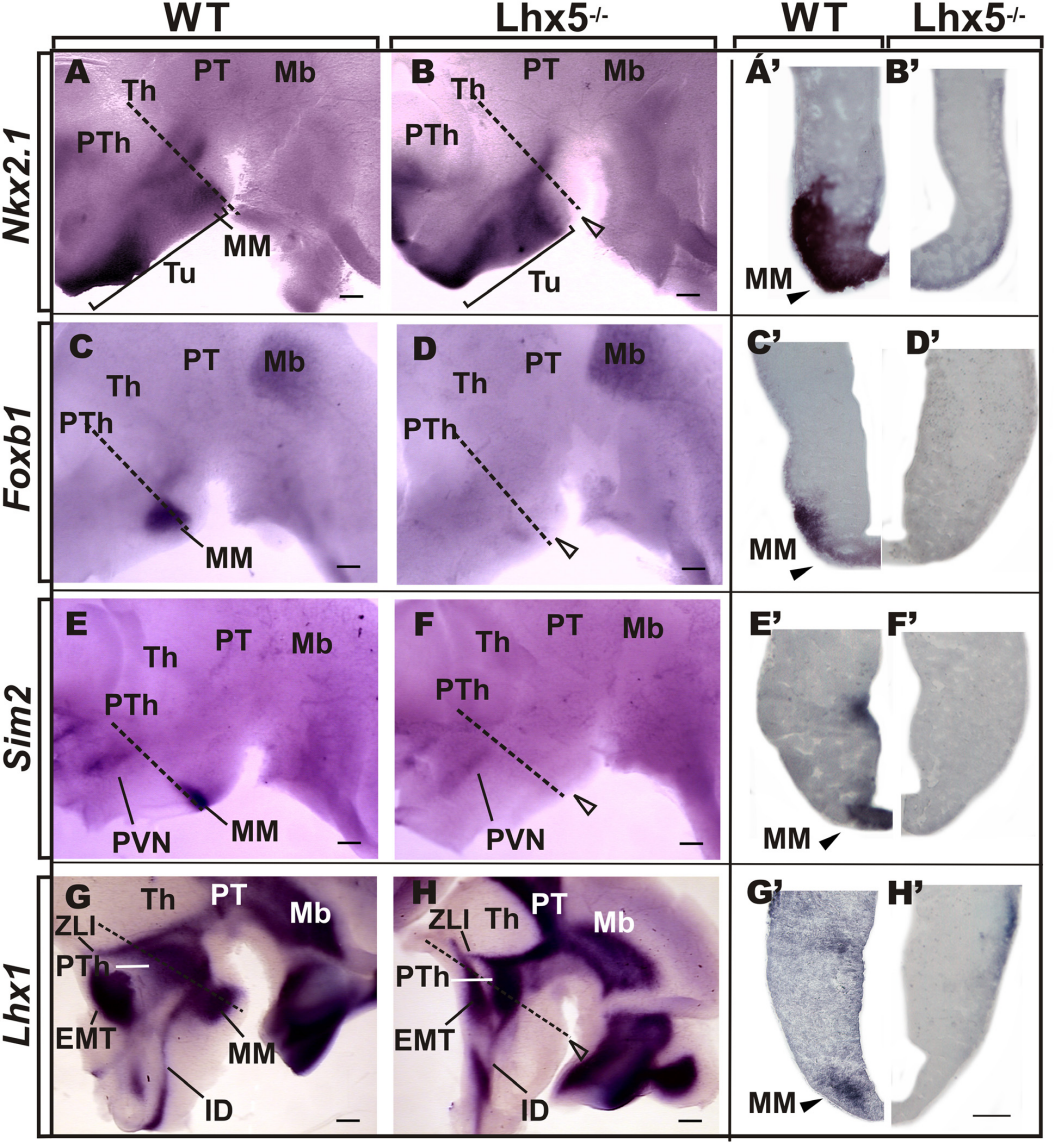
**Comparative analysis of MM markers in the developing embryo.** Genetic expression profile of *Nkx2.1, Foxb1*, *Sim2*, and *Lhx1* in the prospective MM in E12.5 preparations of whole-mount **(A–H**, anterior is to the left) and coronal sections **(A’–H’)** of corresponding Lhx5 mutant and control embryos, as indicated. Empty arrowheads point to the approximate location of MM in Lhx5 mutants. Dashed lines in **(A–H)** indicate the approximate plane of sections shown in **(A’–H’)**. Brackets in **(A,B)** demarcate the extent of *Nkx2.1* expression along the anterio-posterior axis. Abbreviations: Th, thalamus; EMT, eminentia thalami; IDv, ventral intrahypothalamic diagonal; Mb, midbrain; MM, mamillary; PVN, paraventricular nucleus; PT, pretectum; PTh, prethalamus; Tu, tuberal; ZLI, zona limitans intrathalamica. Scale bars: **(A–H)** 400 μm, **(A’–H’)** 200 μm.

First, we compared Nissl-stained sagittal sections of Lhx5 knock-out mice and control littermates, using the pontine gray as anatomical landmark (**Figure 2A**). Whereas a group of neurons in control embryos can be detected at the presumptive MM location (Allen Brain Reference Atlas; Alvarez-Bolado et al., 2000), the mutant posterior hypothalamus displays clear cytoarchitectural alterations and seems to lack a defined MM (**Figure 2B**). We then analyzed the anatomical configuration of axonal bundles in the posterior hypothalamus by Sevier–Munger staining, a silver-based method traditionally used to highlight nerve fibers. During development, mtt axons emerge from the mteg near the boundary between the dorsal and ventral thalami in a Pax-6-dependent manner (Alvarez-Bolado et al., 2000; Valverde et al., 2000). Consistent with the lack of MM, both mtt and mteg branching tracts (readily identifiable at E18.5 in control conditions) could not be found in Lhx5^-/-^ mice (**Figures 2C,D**). Moreover, the afferent fornix tract, which connects the hippocampus to the mamillary region, was absent at comparable regions in *Lhx5* mutants. To further investigate whether *Lhx5* mutants truly lacked MM neurons and if the apparent absence of the structure was not a consequence of altered cytoarchitecture, we performed *in situ* hybridization using MM molecular markers. The expression of *Foxb1*, a bona fide MM marker (Alvarez-Bolado et al., 2000; Szabo et al., 2009a; Shimogori et al., 2010), and of *Lhx1*, a member of the LIM-HD family closely related to *Lhx5* (Bachy et al., 2001; Shimogori et al., 2010), clearly demarcated the MM in control embryos (**Figure 2E**). However, expression of both *Foxb1* and *Lhx1* in the posterior hypothalamus was completely lost in Lhx5 mutants (**Figures 2E–H**). On the other hand, expression of *Lhx1* in the zona incerta and in the suprachiasmatic area (Szabo et al., 2009a), seemed unaffected by the lack of *Lhx5* (**Figures 2G,H**, **3G,H** and data not shown), suggesting that expression of Lhx5 is particularly required for posterior hypothalamic development.

### Early Specification of the MM Requires *LHX5* Function

Since we did not find increased apoptotic cell death in the mutant diencephalon at E12.5 (not shown) and lack of Lhx5 does not impair hypothalamic proliferation (Heide et al., 2015), we asked whether the lack of MM was the result of altered specification of the mamillary area (MM) during early hypothalamic development. To answer this, we performed ISH on E12.5 whole-mount preparations and coronal sections, and compared the expression of genetic markers that label specific regions of the developing hypothalamus (**Figure 3**). At E12.5, the homeodomain transcription factor *Nkx2.1* is expressed throughout the basal hypothalamic area, including the MM (**Figure 3A**) as described previously (Puelles et al., 2000; Szabo et al., 2009a; Shimogori et al., 2010). Analysis of coronal sections revealed *Nkx2.1* in the entire hypothalamic thickness spanning ventricular and mantle regions (**Figure 3A’**). Although *Nkx2.1* expression persisted in the *Lhx5*^-/-^ hypothalamus, its expression domain was dramatically reduced along the anterio-posterior axis and was absent from the MM (**Figures 3B,B’**). Furthermore, expression of the bona fide MM markers *Foxb1*, *Sim2*, and *Lhx1* (Wehr et al., 1997; Alvarez-Bolado et al., 2000; Bachy et al., 2001; Marion et al., 2005; Szabo et al., 2009a; Shimogori et al., 2010; Wolf and Ryu, 2013) was completely absent in Lhx5^-/-^ at comparable regions (**Figures 3C–H,C’–H’**). *Lhx5* mutant embryos lacked the nascent principal mamillary (pm) tract, normally identified by *Foxb1* expression at this stage (**Figures 3C,D**), (Zhao et al., 2007a). In control slices, we detected strong *Foxb1*, *Sim2*, and *Lhx1* expression in mantle regions of the MM, with *Sim2* also present in the ventricular zone, as described elsewhere (**Figures 3C**’–H’), (Marion et al., 2005; Zhao et al., 2008; Shimogori et al., 2010). Notably, lack of *Lhx5* abolished expression of MM markers in both mitotic and post-mitotic compartments. Other hypothalamic regions, however, seemed correctly specified in Lhx5 mutants at the stages analyzed. These include the *Sim2*^+^ paraventricular hypothalamic area (PVN; **Figures 3E,F**) and the anterior part of the intrahypothalamic diagonal (ID), recently identified by *Lhx1* expression (**Figures 3G,H**), (Marion et al., 2005; Shimogori et al., 2010). In addition, the EMT, identified by *Lhx1* and *Tbr1* expression (Retaux et al., 1999; Puelles et al., 2000; Szabo et al., 2009a; Shimogori et al., 2010), seemed to be unaffected by the lack of *Lhx5* (**Figures 3G,H** and data not shown).

**FIGURE 4:**
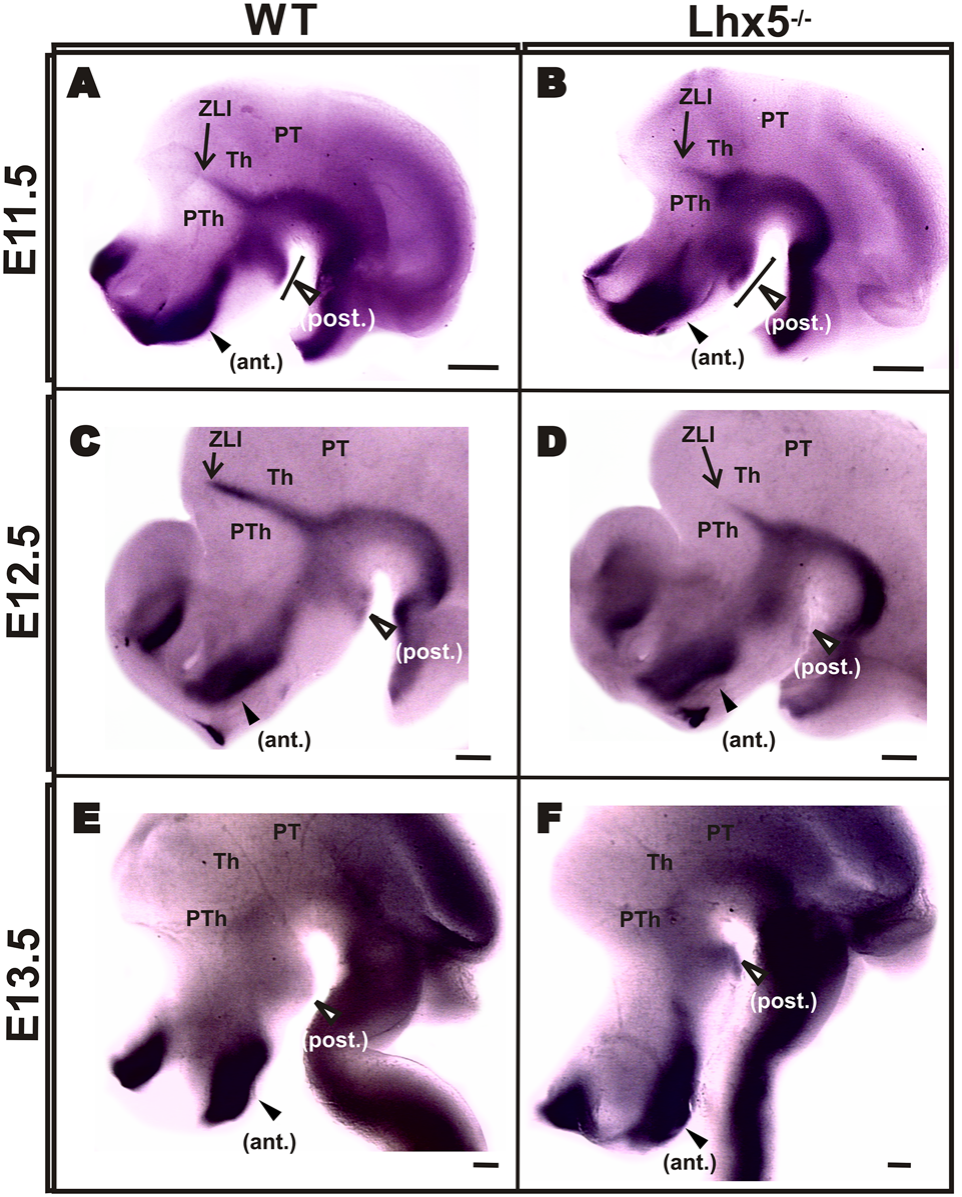
***Shh* expression in Lhx5 mutants.** Comparison of Shh expression on whole-mount ISH in control and mutant embryos at E11.5 **(A,B)**, E12.5 **(C,D)** and E13.5 **(E,F)**. Note that the posterior expression domain (empty arrowheads) is present at all stages analyzed **(A–F)** but appears expanded anteriorly in the mutant at E11.5 (brackets in **A,B**). **(A–D)** The ZLI is present at E11.5 and E12.5, but its dorsal extent (indicated with arrows) is shorter in Lhx5 mutants at E12.5. **(A–F)**. Anterior is to the left. Abbreviations: ant., anterior; Th, thalamus; PT, pretectum; PTh, prethalamus; post., posterior; ZLI, zona limitans intrathalamica. Scale bars: **(A,B)** 200 μm, **(C-F)** 400 μm.

Since Shh signaling is essential for the early regionalization of diencephalic structures (Kiecker and Lumsden, 2004; Scholpp et al., 2006; Shimogori et al., 2010) and for the sustained expression of *Foxb1* in the MM (Szabo et al., 2009a), we sought to investigate whether the defects we observed in the *Lhx5^-/-^* posterior hypothalamus were caused by alterations in *Shh* expression. At the stages analyzed (E11.5–13.5), Shh expression is highly dynamic (Szabo et al., 2009a). In agreement with previous reports, we found strong *Shh* expression in basal and posterior domains of the hypothalamus including MM and tuberal regions, as well as in the ZLI (**Figures 4A,C**), (Szabo et al., 2009a; Shimogori et al., 2010). Although the *Shh* pattern of expression was broadly preserved in Lhx5 mutants, we observed an anterior expansion of the posterior *Shh*^+^ domain at E11.5, in a region that included the MM (**Figures 4A–D**). Along with this expansion, the *Shh*-negative region between the posterior and basal *Shh*^+^ domains was reduced (**Figures 4A,B**). Later, at E12.5 and E13.5, the hypothalamic expression of *Shh* appeared normal in control and Lhx5 mutants (**Figures 4C–F**). The *Shh*-expressing ZLI, however, was shorter on its dorsal reach and thinner in Lhx5 mutants than in littermates at E12.5 (**Figures 4A–D**); a phenotype that was paralleled by similar changes in the *Lhx1* expression domain in this region (**Figures 3G,H**), (Bachy et al., 2001).

Taken together, our results suggest that *Lhx5* is crucial for the specification of the MM from early developmental stages and might function upstream of important region-specific transcription factors such as *Foxb1*, *Sim2*, and *Lhx1*.

### Abnormal Expression of *LHX2* and *LHX9* in the LHX5^-/-^ Hypothalamus

The expression pattern of LIM-HD transcription factors in the embryonic hypothalamus demarcates precise developmental compartments (Shimogori et al., 2010). Based on sequence homology, expression pattern and functional analyses, it has been suggested that the transcription factor pairs Lhx1/5 and Lhx2/9 encode positional information in complementary and antagonistic manners (Retaux et al., 1999; Bachy et al., 2001).

**FIGURE 5:**
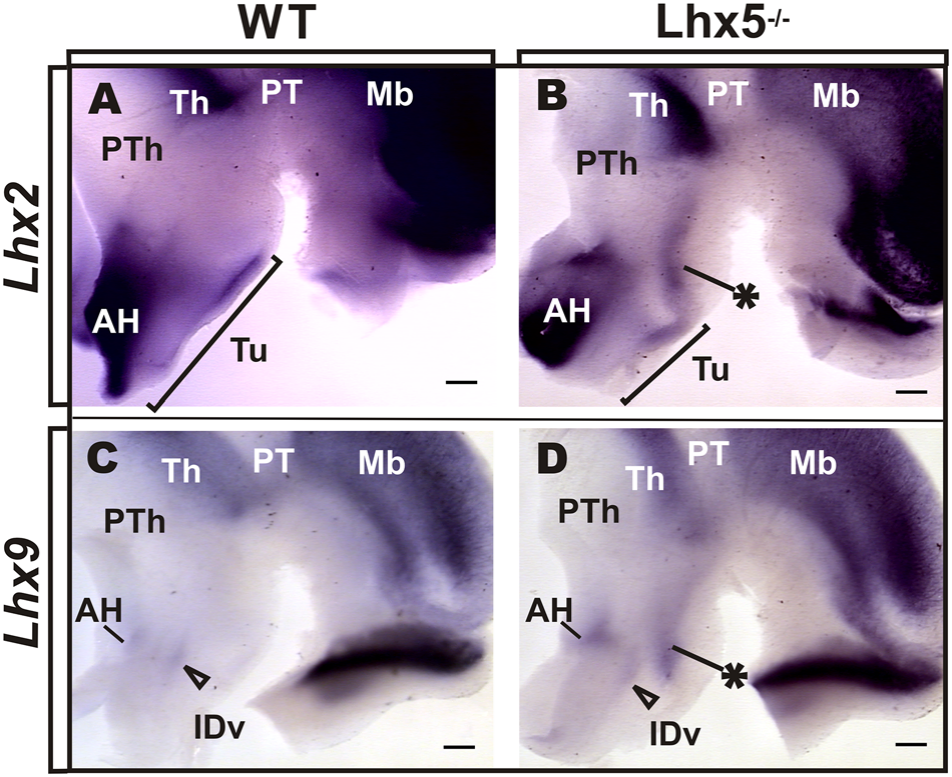
***Lxh2* and *Lhx9* misexpression in the Lhx5^-/-^ posterior hypothalamus.** Whole-mount ISH for *Lhx2* **(A,B)** and *Lhx9* **(C,D)** in control and Lhx5 mutant E12.5 embryos (anterior is to the left), showing ectopic expression of both markers in the posterior hypothalamus of Lhx5 mutants (asterisks in **B,D**). **(A,B)** The *Lhx2*-expressing region in the AH was present in both conditions, but the tuberal expression domain appeared shortened along the anterior–posterior axis (brackets in **A,B**). **(C,D)** *Lhx9* is expressed at low levels in the anterior hypothalamus in both controls and Lhx5 mutants, in a region that might correspond to the IDv domain (empty arrowheads). Note that expression of both genes in the Th seems unaltered in Lhx5 mutants. Abbreviations: AH, anterior hypothalamus; Th, thalamus; IDv, ventral intrahypothalamic diagonal; Mb, midbrain; PT, pretectum; PTh, prethalamus; Tu, tuberal. Scale bar: 200 μm.

We therefore investigated whether the expression of Lhx2 and Lhx9 was altered in the Lhx5 mutant diencephalon at E12.5 (**Figure 5**). We found that both *Lhx2* and *Lhx9* were strongly expressed in the thalamus (Th) and epithalamus in prosomere (p2), as previously described (**Figure 5** and data not shown), (Retaux et al., 1999). In the wild-type hypothalamus, broad expression of *Lhx2* was detected in anterior and tuberal regions, showing a caudally decreasing gradient (**Figure 5A**). In contrast, *Lhx9* appeared almost absent in control embryos, except for a small region in the anterior hypothalamus that may include the ventral domain of the intrahypothalamic diagonal (IDv) that gives rise to Hypocretin (Hcrt)- and possibly Galanin (Gal)-expressing neurons (**Figures 5C,D**), (Shimogori et al., 2010; Liu et al., 2015). Notably, the expression of *Lhx2* and *Lhx9* appeared similarly affected in the *Lhx5*^-/-^ hypothalamus. Ectopic expression domains of both genes appeared in *Lhx5*-expressing territories flanking the dorsal border of the tuberal region with an extension into its ventral region (**Figures 5B,D**). This dorsal domain extended further dorsally toward the *Lhx2/9*^+^ Th and might correspond to an expanded ID. In contrast, *Lhx2* and *Lhx9* expression in the anterior hypothalamic area and thalamus, appeared unaffected in *Lhx5* mutants at this stage. Thus, absence of *Lhx5* induces ectopic expression of *Lhx2/Lhx9* at the presumptive alar/basal diencephalic boundary. Overall, these findings suggest that in addition to specifying the MM, *Lhx5* could play crucial roles in the regionalization of the tuberal hypothalamus by negatively regulating the expression of *Lhx2/Lhx9.*

## Discussion

In this study, we uncovered a key role for the transcription factor Lhx5 in the early patterning of the posterior hypothalamus and formation of the MM.

We found the MM and its associated tracts do not form in the absence of Lhx5. At the end of the gestation period, the MM and its efferent axonal bundles, including the pm, mtt, and mteg that were readily distinguishable in control conditions, could not be detected in Lhx5 mutant mice. Similarly, the afferent fornix tract, which connects the hippocampus to the MM and elongates longitudinally along the caudal aspect of the intrahypothalamic boundary (Puelles and Rubenstein, 2015), was absent in Lhx5 mutants. To confirm the absence of MM neurons in Lhx5 mutants, we analyzed the expression of the bona fide MM markers *Foxb1* and *Lhx1*. Whereas *Lhx1* persisted in anterior hypothalamic regions, both *Foxb1* and *Lhx1* were missing in the prospective Lhx5^-/-^ MM.

Our results suggest that Lhx5 contributes to the MM specification by regulating the expression of key genetic factors that control different aspects of the development of the mamillary region. *Foxb1*, *Sim2*, and *Lhx1* are commonly used to identify the mamillary region from early developmental stages (Wehr et al., 1997; Alvarez-Bolado et al., 2000; Marion et al., 2005; Zhao et al., 2008; Szabo et al., 2009a; Shimogori et al., 2010). At E12.5, we found clear labeling for these markers of the MM in controls, but they were completely absent in the prospective MM of Lhx5 mutants. Important roles for *Sim2* and *Foxb1* have been reported in the formation of MM tracts. Specifically, *Foxb1* is needed for the formation of the mtt, whereas both *Sim1* and *Sim2* are synergistically required for mtt and mteg development (Alvarez-Bolado et al., 2000; Marion et al., 2005). Interestingly, *Foxb1* is also needed to maintain the medial component of the MM in adulthood, despite the fact that its expression is restricted to embryonic stages (Wehr et al., 1997; Zhao et al., 2008). Compound Sim1/Sim2 knock-out mice show decreased levels of *Foxb1* and unaltered *Lhx1* expression, suggesting that Sim1/2 act upstream of *Foxb1* but do not control *Lhx1* (Marion et al., 2005). In the current study, we found *Lhx1* to be completely absent from the MM of Lhx5 mutants, which indicates that Lhx5 drives *Lhx1* expression in this area. This is supported by our previous observation of *Lhx1* downregulation in the Lhx5^-/-^ telencephalon (Miquelajauregui et al., 2010). To our knowledge, this is a first example of MM ablation by genetic means from early developmental stages. In keeping with these results, in an accompanying paper we describe the molecular mechanisms underlying the MM disappearance after initial specification using an Lhx5 hypomorphic allele (Heide et al., 2015).

Consistent with the lack of MM marker expression in Lhx5 mutants, we found an anterior shift in the expression of the general basal hypothalamic marker *Nkx2.1* and its apparent absence in the posterior area corresponding to the MM. It has been previously shown that Nkx2.1 is required for the specification of the ventrobasal hypothalamus, including the MM. The absence of Nkx2.1 function leads to decreased *Sim1* expression in the posterior hypothalamus and a general ventral-to-dorsal shift in its molecular properties (Kimura et al., 1996). Altered Slit/Robo signaling has been associated with abnormal hypothalamic patterning in Nkx2.1 and Sim1/2 mutants (Marin et al., 2002; Marion et al., 2005). We deem it unlikely that the effects of Lhx5 disruption are mediated by Slit/Robo signaling, as expression of several members of these families persisted in the Lhx5^-/-^ hypothalamus (data not shown).

We also asked whether Shh expression was affected in the mutant diencephalon, as this morphogen is an important regulator of diencephalic regionalization known to exhibit a complex expression pattern in this region (Manning et al., 2006; Szabo et al., 2009a). We confirmed the presence of two main diencephalic sources of Shh in the developing mouse embryo: one in the basal hypothalamus (strong at both anterior and posterior ends) and the other delineating the ZLI (Shimogori et al., 2010). Notably, we found an expansion of the posterior Shh^+^ domain in the basal hypothalamus of Lhx5 mutants at E11.5. This posteroventral domain might correspond to the region lacking *Nkx2.1* expression a day later at E12.5 (**Figures 3A,B**). Interestingly, it has been shown that sustained expression of Shh signaling eliminates posterior hypothalamic fates, as defined by lack of *Emx2* expression (Manning et al., 2006). Conversely, Shh deletion leads to increased expression of *Emx2* in the posterior hypothalamus (Mathieu et al., 2002; Szabo et al., 2009b). Interestingly, conditional deletion of Shh in embryos carrying the Nkx2.1-Cre and Shh-loxP transgenic alleles, led to strong defects in anterior and tuberal regions but the expression of MM markers was preserved in the posterior hypothalamus (Shimogori et al., 2010). Overall, our data suggest that Lhx5 promotes MM specification via downregulation of Shh in the posteroventral hypothalamus. It is likely that the dynamic expression of Shh and its crosstalk with other signaling pathways, such as those involving BMP, FGF, and Wnt, influence different aspects of posterior hypothalamic development (Manning et al., 2006; Blackshaw et al., 2010). For example, Lhx5 could regulate Wnt signaling in the hypothalamus, as demonstrated by the Sfrp-mediated negative regulation of Wnt signaling by Lhx5 in the zebrafish forebrain (Peng and Westerfield, 2006).

In Lhx5 mutants, we also found altered hypothalamic expression of the closely related LIM homeobox transcription factors *Lhx2* and *Lhx9*. Specifically, we found an ectopic longitudinal domain expressing Lhx2/9 along the anterio-posterior axis, in a region that might overlap with the basal-alar boundary and the ID, recently described as being rich in the expression of LIM-HD members (Shimogori et al., 2010). The anterior hypothalamic *Lhx2* and *Lhx9* expression, however, persisted in Lhx5 mutants, in agreement with the unaltered *Sim2* expression observed in this region. Hence, these findings suggest that in addition to specifying the MM, Lhx5 plays additional roles in the regionalization of basal (e.g., tuberal) and alar hypothalamus (including SPa and ABas domains). In agreement with the observation of highly conserved, mutually exclusive expression patterns of *Lhx2/9* and *Lhx1/5* in the developing diencephalon (Retaux et al., 1999; Bachy et al., 2001), our study suggests that *Lhx2* and *Lhx9* are negatively regulated by Lhx5.

Notably, *Lhx9* is expressed in an *Nkx2.1*-negative region at E12.5 that later overlaps with the postnatal expression of Hcrt and Gal (Shimogori et al., 2010). Although diencephalic development seems to proceed normally in single Lhx9 and Lhx2 knockout mice (Birk et al., 2000; Lakhina et al., 2007), overexpression of *Lhx9* leads to a transient upregulation of Hcrt-expressing neurons (Liu et al., 2015) that may require *Lhx2* and/or additional co-factors for sustained survival. In agreement with this, the simultaneous activation of *Lhx2* and *Lhx9* is required for diencephalic patterning in zebrafish (Peukert et al., 2011).

The hypothalamus is an important region controlling neuroendocrine, physiological and memory functions. Due to their intricate expression pattern, it will be important to dissect the specific roles of LIM homeobox members in hypothalamic development, their dynamic influence on signaling pathways and their precise functional interactions. Lhx5 appears to be part of a complex and dynamic regulatory pathway that patterns posterior hypothalamic regions through the regulation, directly or indirectly, of *Foxb1*, *Sim2*, *Lhx1*, and even *Nkx2.1* in mamillary regions, and *Lhx2* and *Lhx9* in tuberal regions (Blackshaw et al., 2010; Shimogori et al., 2010). It is also possible that Lhx5 negatively regulates other posterior identities, as revealed by the complementary expression of *Lhx5* and the RM markers *Lmx1b* and *Irx5* (Supplementary Figure S1; Ferran et al., 2015).

In this study, perinatal death of Lhx5 mutant mice precluded further behavioral analyses although previous studies detected learning impairments and motor dysfunction related to the absence of Lhx5 (Paylor et al., 2001). Future studies using conditional mutants could enable the identification of behavioral alterations derived directly from the mamillary defects in Lhx5 mutants and to assess the specific roles of the MM in the postulated circuits of memory and spatial navigation.

## Author Contributions

AM and AV-E designed and performed experiments, analyzed data and wrote the manuscript. All other authors participated in acquisition, analysis, or interpretation of data and revised and approved the contents of the manuscript.

## Conflict of Interest Statement

The Associate Editor Dr Puelles declares that, despite having collaborated with author Dr Alvarez-Bolado, the review process was handled objectively. The authors declare that the research was conducted in the absence of any commercial or financial relationships that could be construed as a potential conflict of interest.

## Supplementary material

FIGURE S1 ***Lhx5* and *Lmx1b* expression patterns in the posterior hypothalamus appear negatively correlated.** Allen Brain Atlas (Developing mouse) ISH data from sagittal sections of E11.5 **(A,B)** and E13.5 **(C,D)** wild-type mice (anterior is to the left), comparing the expression pattern of *Lhx5* in the MM and that of *Lmx1b* and *Irx5*, markers of the RM at equivalent medio-lateral levels. Dashed lines delineate the putative alar-basal boundary. Abbreviations: EMT, eminentia thalami; MM, mamillary; RM, retromamillary; SCN, suprachiasmatic nucleus; Tu, tuberal; ZLI, zona limitans intrathalamica. Scale bar: 400 μm.
