# Supplementary figures and images for: LIM homeobox protein 5 (Lhx5) is essential for mamillary body development
Source: Front Neuroanat. 2015 Oct 27;9:136. doi: 10.3389/fnana.2015.00136 (PMC4621302; doi:10.3389/fnana.2015.00136)

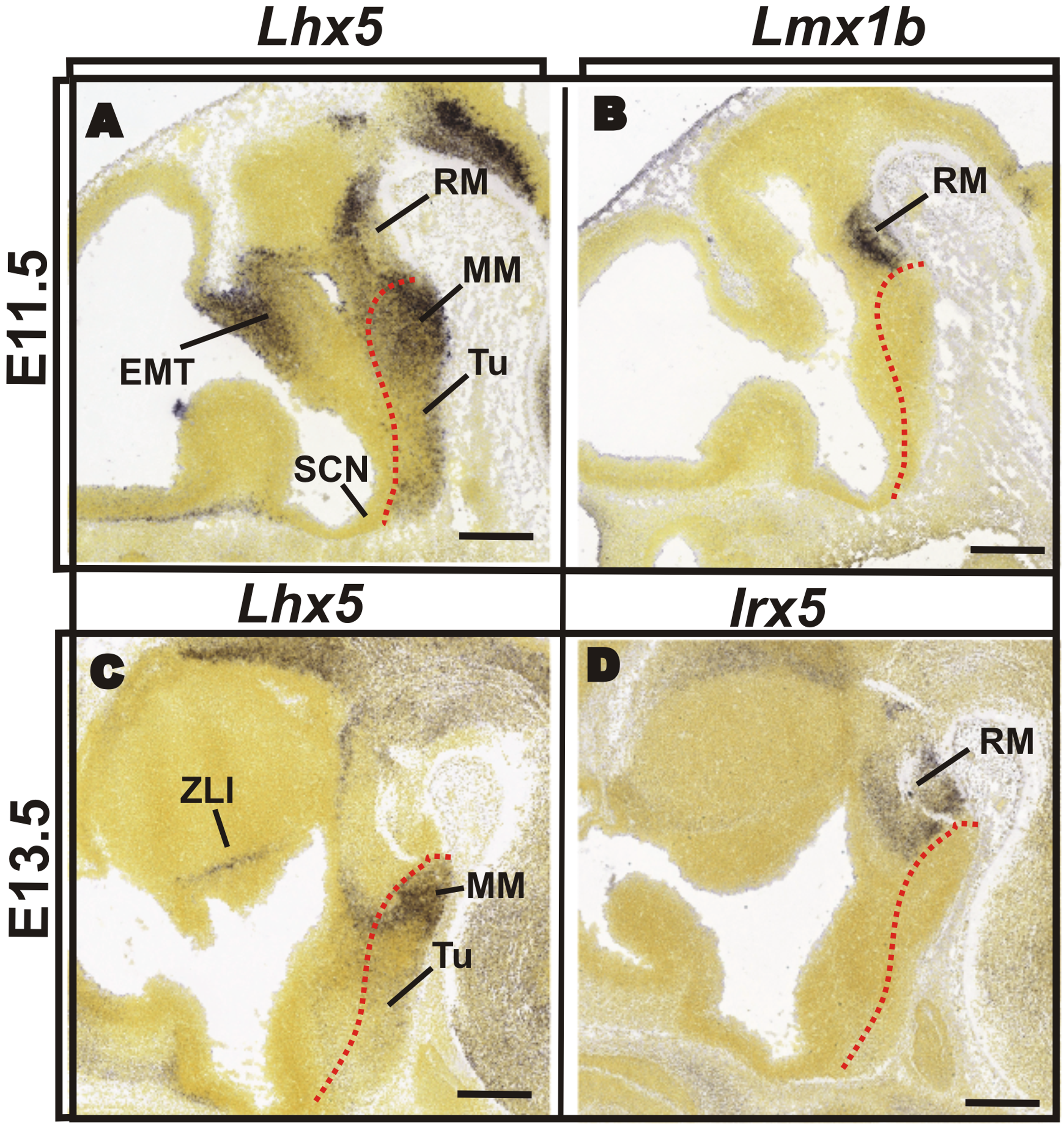

Supplement: FIGURE S1 — Lhx5 and Lmx1b expression patterns in the posterior hypothalamus appear negatively correlated. Allen Brain Atlas (Developing mouse) ISH data from sagittal sections of E11.5 (A,B) and E13.5 (C,D) wild-type mice (anterior is to the left), comparing the expression pattern of Lhx5 in the MM and that of Lmx1b and Irx5, markers of the RM at equivalent medio-lateral levels. Dashed lines delineate the putative alar-basal boundary. Abbreviations: EMT, eminentia thalami; MM, mamillary; RM, retromamillary; SCN, suprachiasmatic nucleus; Tu, tuberal; ZLI, zona limitans intrathalamica. Scale bar: 400 μm. [file Image_1.TIF]
